# Analysis of Oral Health-Related Quality of Life in Elderly Romanian Edentulous Patients: Implant-Supported Versus Conventional Complete Dentures

**DOI:** 10.3390/jcm13226865

**Published:** 2024-11-14

**Authors:** Denisa Tabita Sabău, Abel Emanuel Moca, Raluca Iulia Juncar, Teofana Bota, Mihai Juncar

**Affiliations:** 1 Doctoral School of Biomedical Sciences, University of Oradea, 1 Universității Street, 410087 Oradea, Romania; denisa.sabau@gmail.com (D.T.S.); teofana.bota@gmail.com (T.B.); 2 Department of Dentistry, Faculty of Medicine and Pharmacy, University of Oradea, 10 Piața 1 Decembrie Street, 410073 Oradea, Romania; abelmoca@yahoo.com (A.E.M.); mihaijuncar@gmail.com (M.J.)

**Keywords:** OHRQoL, edentulous patients, implant-supported dentures

## Abstract

**Background/Objectives:** The integration of quality of life (QoL) assessments into dental health evaluations acknowledges the profound impact of oral health on overall well-being. This study aims to compare the impact of implant-supported complete dentures versus conventional complete dentures on oral health-related quality of life (OHRQoL) in elderly Romanian edentulous patients. **Methods:** This longitudinal study involved 93 initially recruited edentulous patients, with 52 completing the study over an 18-month period. Data collection utilized the OHIP-5 questionnaire, assessing the OHRQoL at baseline with conventional dentures and three months post-implant-supported denture placement. Ethical approval was secured from the Faculty of Medicine and Pharmacy University of Oradea, adhering to the Helsinki Declaration principles. A statistical analysis was conducted using SPSS version 25 and included non-parametric tests for score comparisons and Fisher’s exact test for categorical data. **Results:** The comparative analysis of the questionnaire responses revealed significant improvements in all five OHRQoL dimensions post-treatment with implant-supported dentures. For instance, the proportion of patients reporting “never” experiencing difficulty chewing any foods increased from 1.9% at baseline to 57.7% post-treatment. Similarly, those reporting “never” experiencing painful aching rose from 3.8% to 76.9%. There was also a notable reduction in discomfort regarding the appearance of mouth, dentures, or jaws from 3.8% reporting “never” at baseline to 75% post-treatment. The improvements in sense of taste and difficulty in performing usual activities saw comparable increases. **Conclusions:** The findings support the hypothesis that implant-supported complete dentures significantly enhance OHRQoL among elderly edentulous patients compared to conventional dentures, with improvements noted in mastication ability, pain reduction, aesthetics, taste perception, and activity performance. These results underscore the value of prosthetic interventions in dental care to substantially improve patients’ OHRQoL.

## 1. Introduction

The concept of health is multifaceted and lacks a universal tool for its comprehensive measurement. Traditionally, mortality trends, life expectancy, and morbidity indices are employed to describe a nation’s health status [1]. However, these measures often fail to capture the broader dimensions of well-being. In response, quality of life (QoL) assessment techniques have been developed to provide a more holistic evaluation of health [2]. The World Health Organization Quality of Life Assessment Group defines QoL as an individual’s perception of their position in life, contextualized within their cultural setting, value systems, goals, expectations, standards, and concerns [3].

In recent years, there has been a paradigm shift in healthcare from a primary focus on diagnosis and treatment to a broader emphasis on patient-centered care, with QoL emerging as a key consideration [2]. The interrelationship between oral health and overall health is increasingly recognized by both healthcare professionals and patients [4,5,6]. 

OHRQoL refers to the impact of oral health conditions on various aspects of daily life, including physical, psychological, and social well-being [7]. Oral health plays a fundamental role in daily functioning, affecting critical activities such as eating, speaking, and social interactions, as well as influencing self-esteem, overall psychological well-being, and hypersensitivity [8]. Tooth loss significantly impairs the ability to chew, leading to dietary restrictions and potential nutritional deficiencies. The Oral Health Impact Profile (OHIP) is one of the most widely used tools for assessing OHRQoL, evaluating dimensions such as function, pain, physical and social disability, and handicap through 49 standardized items [9].

Over time, several shorter versions and translations of the OHIP have been developed to enhance practicality while maintaining reliability. One such version is the OHIP-5, a condensed form comprising five questions, which has been shown to reliably capture 86% of the variability in OHRQoL measured by the original OHIP-49 [10,11]. The OHIP-5 is particularly advantageous in clinical and research settings due to its brevity and ease of use, allowing for the efficient yet comprehensive assessment of OHRQoL across diverse oral health conditions [12,13,14,15].

Understanding patients’ subjective experiences is crucial, as their perceptions of disease and treatment often have a more significant effect on their QoL than objective clinical indices. There is evidence to suggest a disconnect between patients’ satisfaction with dentures and dentists’ evaluations, highlighting the importance of incorporating patient-reported outcomes into clinical practice [10].

Therefore, this study aims to evaluate and compare the impact of implant-supported complete dentures and conventional complete dentures on the OHRQoL among Romanian elderly edentulous patients. By analyzing improvements in key quality-of-life indicators—including mastication ability, comfort, aesthetics, taste perception, and ease of performing daily activities—this research seeks to provide a clearer understanding of how implant-supported dentures may offer enhanced therapeutic benefits over conventional options. Additionally, this study addresses the broader implications of denture choice on patient satisfaction and overall well-being, contributing valuable insights for clinical decision-making in prosthetic dentistry.

## 2. Materials and Methods

### 2.1. Ethical Considerations

This study was approved by the Ethics Committee of the Faculty of Medicine and Pharmacy, University of Oradea (IRB No. CEFMF/1, dated 13 April 2023) and was conducted in accordance with the principles of the 2008 Helsinki Declaration and its subsequent amendments. The participants were informed that their involvement in this study was voluntary and that their identity would remain confidential. Although names were required for the administration of the study, they would not be disclosed at any point. Informed consent was obtained from all the participants, which was evidenced by their voluntary completion of the questionnaires at two distinct assessment points.

### 2.2. Participants and Data Collection

This longitudinal study was conducted over an 18-month period, from 1 March 2023 to 1 September 2024, and included patients with complete monomaxillary or bimaxillary edentulism who sought examination and treatment at a dental clinic in Oradea, Romania. Participants were recruited from the clinic’s patient pool during their initial examination. Some patients were referred by their primary dentists, while others self-referred for consultation and treatment. Based on patient history, tooth loss was primarily attributed to complications of caries and periodontal disease. The initial examinations were conducted by two of the authors, both of whom have over 15 years of experience and are specialists in Dental Prosthetics.

The data were collected using questionnaires administered in two phases through the online platform Survio (Survio s.r.o., Brno, Czech Republic), a tool specifically designed for conducting online surveys. Following the initial clinical assessment, which confirmed complete monomaxillary or bimaxillary edentulism and evaluated the quality of the patient’s conventional denture, a link to the first questionnaire (Q1) was sent to the patient’s mobile phone. A total of 93 patients received the initial questionnaire.

The second questionnaire (Q2) was distributed three months after the patients received dental implants (4, 6, or 8 dental implants per arch) and fixed implant-supported dentures with multiunit abutments (MUAs). All implants used were Implant Swiss (Novodent SA, Yverdon-les-Bains, Switzerland), featuring surfaces treated with the Double Acid Etching technique to improve osseointegration. Of the 93 patients who completed the initial survey, 52 responded to the follow-up questionnaire. Only those who completed the initial questionnaire were invited to participate in the follow-up phase.

This study employed a modified version of the OHIP-49 questionnaire, referred to as OHIP-5 [12], which is an ultra-short instrument designed for the rapid assessment of oral health-related quality of life. The questionnaire was translated into Romanian and consisted of five questions specifically adapted for individuals using either conventional complete dentures or implant-supported prostheses:

- Item 1: Have you had difficulty chewing any foods because of problems with your mouth, dentures, or jaw?
- Item 2: Have you experienced painful aching in your mouth?
- Item 3: Have you felt uncomfortable about the appearance of your mouth, dentures, or jaws?
- Item 4: Have you noticed a reduced sense of taste due to problems with your mouth, dentures, or jaws?
- Item 5: Have you had difficulty performing your usual activities due to problems with your mouth, dentures, or jaws?

The participants selected their responses using a 5-point Likert scale, with options ranging from Never (1 point) to Very Often (5 points). This scoring system allowed for a quantitative analysis of oral health-related quality of life.

The demographic data collected included only age and gender, which were used to characterize the study population and to assess potential variations in responses based on these factors.

The participants included in this study were those diagnosed with complete edentulism in either a single arch (upper or lower) or both arches. All the participants were required to be Romanian-speaking individuals to ensure effective communication and understanding of the study procedures. Additionally, they had to express an interest in receiving implant-supported dentures. Only those with the cognitive and psychological capacity to effectively communicate and fully participate in this study were considered eligible.

The exclusion criteria encompassed participants who, at the time of the initial examination, already had an implant-supported denture or had conventional dentures older than one year. Smokers and patients who did not wish to receive an implant-supported denture were also excluded. Additional criteria encompassed incomplete responses to the initial questionnaire, non-Romanian speakers, and individuals with medical conditions contraindicating dental implant surgery. Patients with cognitive impairments or psychological conditions impacting their ability to fully understand or adhere to study procedures were also excluded to ensure the validity of the study results and compliance with protocol requirements.

### 2.3. Statistical Analysis

The statistical analysis was conducted using IBM SPSS Statistics version 25 (IBM, Chicago, IL, USA), with data visualization performed in Microsoft Office Excel and Word 2021 (Microsoft, Redmond, WA, USA). The distribution of quantitative variables was assessed using the Shapiro–Wilk test. These variables were presented as means with standard deviations or as medians with interquartile ranges, depending on their distribution. Independent quantitative variables exhibiting a non-parametric distribution were compared between groups using the Mann–Whitney U test or the Kruskal–Wallis H test, as appropriate.

The qualitative variables were reported as absolute counts and percentages. The differences between the groups were evaluated using Fisher’s exact test. To further explore significant findings from the contingency tables, Z-tests with Bonferroni correction were employed for multiple comparisons.

## 3. Results

The final sample consisted of 52 patients who completed the OHIP-5 questionnaire on two occasions: initially, when they were already fitted with a conventional denture (Q1), and subsequently, after receiving an implant-supported denture (Q2). The demographic breakdown included 25 female patients (48.1%) and 27 male patients (51.9%). The mean age of the participants was 60.56 ± 9.26 years at the time of the first questionnaire and 62.06 ± 9.26 years at the second. In this study, 23 patients (44.23%) received four implants per arch, another 23 patients (44.23%) received six implants per arch, and 6 patients (11.54%) received eight implants per arch.

### 3.1. Comparative Analysis of Questionnaire Responses

The data presented in Table 1 detail the comparative analysis of the scores for the five items at two different assessment points: the initial examination (Q1) and three months after receiving an implant-supported denture (Q2). The score distribution for all five items was non-parametric in both treatment phases as determined by the Shapiro–Wilk test (*p* < 0.001), and inter-group differences were statistically significant according to the Mann–Whitney U test (*p* < 0.001). The results showed the following:

- For Item 1, “Have you had difficulty chewing any foods because of problems with your mouth, dentures, or jaw?”, the patients reported significantly higher difficulties in mastication during Q1 than in Q2.
- For Item 2, “Have you experienced painful aching in your mouth?”, the responses indicated that the patients experienced more painful aching in the mouth in Q1 compared to Q2.
- For Item 3, “Have you felt uncomfortable about the appearance of your mouth, dentures, or jaws?”, there was a significant increase in discomfort regarding the appearance of their mouth, dentures, or jaws in Q1 compared to Q2.
- For Item 4, “Have you noticed a reduced sense of taste due to problems with your mouth, dentures, or jaws?”, the patients noted a significantly reduced sense of taste in Q1 as opposed to Q2 due to issues with their mouth, dentures, or jaws.
- For Item 5, “Have you had difficulty performing your usual activities due to problems with your mouth, dentures, or jaws?”, the scores suggest that the patients faced more difficulties performing usual activities due to oral health issues in Q1 than in Q2.

Table 2 provides supplementary data on the distribution of responses for the five evaluated items, comparing outcomes between patients with conventional dentures (Q1) and those with implant-supported dentures (Q2). The statistical analysis employed the Fisher exact test, revealing significant differences (*p* < 0.001) for all the items, which further supports the improvements noted in Table 1, such as the following:

- For Item 1, in Q2, a higher proportion of the patients reported “Never” (57.7% vs. 1.9%) or “Almost Never” (25% vs. 3.8%) experiencing difficulties in chewing, contrasting with Q1, where more patients reported frequent difficulties (Often: 42.3% vs. 1.9%; Very Often: 21.2% vs. 0%).
- For Item 2, a significant shift was observed with 76.9% of the patients in Q2 “Never” experiencing sharp pains, compared to only 3.8% in Q1. Conversely, “Occasional” pain was more commonly reported in Q1 (57.7% vs. 3.8%).
- For Item 3, responses indicating “Never” feeling uncomfortable about dental appearance were significantly higher in Q2 (75% vs. 3.8%). In contrast, reports of “Occasional” (38.5% vs. 7.7%), “Often” (30.8% vs. 0%), and “Very Often” (13.5% vs. 0%) discomfort were more prevalent in Q1.
- For Item 4, the majority of patients in Q2 reported “Never” experiencing a reduced sense of taste (92.3% vs. 1.9%), whereas “Occasional” (15.4% vs. 1.9%), “Often” (44.2% vs. 0%), and “Very Often” (34.6% vs. 0%) responses were more common in Q1.
- For Item 5, in Q2, a significant number of patients indicated they “Never” faced difficulties in carrying out activities due to dental problems (78.8% vs. 3.8%). However, “Occasional” (51.9% vs. 3.8%), “Often” (25% vs. 0%), and “Very Often” (9.6% vs. 0%) difficulties were more frequently reported in Q1.

### 3.2. Influence of Gender and Age on Questionnaire Responses

The investigation into the influence of gender on the responses to the five items revealed that gender did not significantly affect the outcomes. The statistical analysis using the Fisher exact test showed no significant differences between the male and female patients for any of the five items (Table 3). Thus, the responses to both the Q1 and Q2 questionnaires were consistent across genders.

Further analysis explored the potential influence of age on the responses provided for the five items. The results indicated that age did not significantly impact the responses, as the statistical analysis revealed no significant differences between age groups in either Q1 or Q2 (Table 4). This finding suggests that the improvements noted after transitioning to implant-supported dentures were consistent across different age groups.

## 4. Discussion

This study evaluated the impact of 3 months post-implant-supported complete dentures compared to conventional complete dentures on OHRQoL among elderly edentulous patients. For conventional complete denture wearers, achieving occlusal stability can be challenging due to the lack of underlying anchorage [16]. These cases may benefit from simpler occlusal designs to avoid excessive pressure and discomfort, minimize frontal forces, and prevent prosthesis dislodgment by optimizing force distribution along the alveolar ridge [16]. In contrast, implant-supported dentures significantly enhance stability due to the implants, which allow for greater flexibility in occlusal design. More complex occlusal schemes can be utilized, improving the coordination of masticatory muscles and optimizing orofacial function [17]. Balanced occlusal schemes, which distribute forces evenly, may be particularly suitable as they maximize the functional surface area, enhancing masticatory efficiency and patient comfort [18]. Implant-supported complete dentures offer a closer approximation to natural dentition, resulting in a stronger, more stable bite force and improved masticatory efficiency [19]. Interestingly, patients wearing conventional dentures tend to seek dental care only when their prosthesis becomes unusable, and clinically unsatisfactory dentures have the potential to negatively impact the patient’s quality of life [20]. A viable alternative to mitigate the potential issues associated with conventional dentures is implant-supported dentures, which provide significantly better stability and offer psychosocial, functional, and biological benefits for patients [21].

The findings of our study demonstrate significant improvements in all five OHRQoL dimensions following the placement of implant-supported dentures, confirming the hypothesis that these prostheses significantly enhance patients’ quality of life. The most notable improvements were observed in the domains of mastication, pain, and aesthetics. 

Prior to receiving implant-supported dentures, only 1.9% of the participants reported “Never” experiencing difficulty chewing any foods, which dramatically increased to 57.7% post-treatment. This suggests that implant-supported dentures effectively restore functional capabilities, aligning with previous studies highlighting the superiority of implant prosthetics in masticatory efficiency and patient satisfaction compared to conventional dentures. Filius et al. (2018) monitored the effect of implant-supported fixed prosthodontics in a cohort of patients from the Netherlands and found a significant decrease in OHIP scores after treatment with dental implants, indicating an improved OHRQoL post-treatment [22]. Similarly, Coltro et al. (2022) evaluated OHRQoL levels in a cohort of patients after treatment with implant-supported fixed complete dentures using the OHIP-14, identifying a long-term positive effect on patient quality of life [23]. In our study, in addition to analyzing individual responses before (Q1) and after treatment with implant-supported dentures (Q2), the OHIP-5 questionnaire scores significantly decreased in Q2 compared to Q1 across all five items.

Similarly, the reduction in painful aching in the mouth—from 3.8% of participants reporting “Never” at baseline to 76.9% post-treatment—underscores the role of implant-supported dentures in minimizing oral discomfort. Orofacial pain is one of the most widespread health issues that can profoundly affect a patient’s quality of life and has a significant social impact [24]. This finding is consistent with the existing literature, which suggests that conventional dentures can exacerbate mucosal soreness and discomfort due to their limited retention and stability [25], whereas implants provide a more secure fit, reducing irritation [26]. Sivaramakrishnan and Sridharan (2016) similarly reported a reduction in perceived pain levels following treatment with implant-supported dentures [27].

The aesthetic and psychosocial dimensions also saw significant improvements, with 75% of the patients post-treatment indicating they “Never” felt uncomfortable about the appearance of their mouth, compared to only 3.8% at baseline. The psychological impact of improved aesthetics, including enhanced self-esteem and social confidence, has been well documented, supporting the view that dental implants contribute not only to physical health but also to emotional and social well-being [28,29]. McCrea (2017) identified a significant statistical relationship between implant-supported prosthetic reconstruction and patients’ overall satisfaction with their appearance [29]. Similarly, Bajunaid et al. (2022) reported higher levels of satisfaction among patients with implant-supported dentures [30]. In our study, scores for the item “Have you felt uncomfortable about the appearance of your mouth, dentures, or jaws?” significantly decreased in Q2, indicating an increase in self-esteem and comfort with the appearance of the oral cavity post-treatment.

Interestingly, the improvements in taste perception and ease of performing usual activities, while significant, were less pronounced than in other domains. This may be attributed to the adaptation period required for patients to fully acclimate to their new dentures, particularly in relation to changes in oral sensation and the coordination required for certain activities [31]. However, studies indicate that patient satisfaction in these areas tends to increase over time. Neshandar Ali et al. (2021) evaluated short-term patient satisfaction at one month and three months post-treatment and observed a significant increase in satisfaction at these intervals [32]. This trend of adaptation was also identified at a five-year follow-up [33].

This study’s findings contribute to the growing body of evidence supporting the integration of patient-reported outcomes in dental care. The discrepancy between clinical assessments of denture fit and patient satisfaction has been well documented [34], and the present study reaffirms the importance of considering subjective experiences when evaluating treatment success. The use of the OHIP-5 questionnaire provided a concise yet comprehensive measure of OHRQoL, demonstrating robust psychometric properties even in a shortened format [12]. 

There were no significant differences in OHRQoL outcomes based on gender or age, suggesting that the benefits of implant-supported dentures are consistent across demographic groups within the study sample. This finding is encouraging, as it indicates that elderly patients of varying ages and genders can equally benefit from this advanced prosthetic treatment.

Several limitations should be acknowledged. This study’s relatively small sample size and specific demographic focus may limit the generalizability of the findings. Additionally, the follow-up period was restricted to three months post-implant placement, which may not capture long-term outcomes or complications associated with implant-supported dentures. Another limitation of this study is the inclusion of both fully edentulous patients and those with single-arch edentulism without separate analysis or stratification by these groups. This approach may introduce variability, as the presence of natural teeth in one arch could affect the experience and functionality of removable or implant-supported dentures. Moreover, the reliance on self-reported measures may introduce bias, as patient perceptions can be influenced by factors not directly related to the prosthetic treatment itself. In addition to this, there was a variability in the number of implants used per patient, with some receiving four, others six, and others eight implants per arch. This variation was guided by individual anatomical and clinical requirements to ensure adequate stability and functionality. However, this lack of uniformity in implant count introduces a level of heterogeneity that may affect the comparability of outcomes across patients. Future studies with a standardized implant protocol would allow for more controlled conditions and could further clarify the specific impact of implant quantity on therapeutic outcomes and orofacial function.

## 5. Conclusions

This study has demonstrated that implant-supported complete dentures significantly outperform conventional dentures in enhancing the OHRQoL of edentulous patients. The most notable improvements were observed in chewing ability and social confidence. These findings emphasize the transformative impact of implant-supported dentures on both the physical and psychosocial aspects of patients’ lives.

Future research should focus on expanding these findings to diverse populations and exploring long-term outcomes, ensuring that all patients have the opportunity to experience the substantial quality-of-life improvements offered by implant-supported dentures.

## Figures and Tables

**Table 1:** Comparison of scores for Q1 and Q2.

Questionnaire	Mean Score ± SD	Median Score (IQR)	*p* *
**Item 1: Have you had difficulty chewing any foods because of problems with your mouth, dentures, or jaw?**			
Q1 (*p* < 0.001 **)	3.77 ± 0.9	4 (3–4)	<0.001
Q2 (*p* < 0.001 **)	1.62 ± 0.82	1 (1–2)	
**Item 2: Have you experienced painful aching in your mouth?**			
Q1 (*p* < 0.001 **)	2.79 ± 0.75	3 (2–3)	<0.001
Q2 (*p* < 0.001 **)	1.31 ± 0.64	1 (1–1)	
**Item 3: Have you felt uncomfortable about the appearance of your mouth, dentures, or jaws?**			
Q1 (*p* < 0.001 **)	3.37 ± 1.01	3 (3–4)	<0.001
Q2 (*p* < 0.001 **)	1.33 ± 0.61	1 (1–1.75)	
**Item 4: Have you noticed a reduced sense of taste due to problems with your mouth, dentures, or jaws?**			
Q1 (*p* < 0.001 **)	4.06 ± 0.91	4 (4–5)	<0.001
Q2 (*p* < 0.001 **)	1.1 ± 0.35	1 (1–1)	
**Item 5: Have you had difficulty performing your usual activities due to problems with your mouth, dentures, or jaws?**			
Q1 (*p* < 0.001 **)	3.27 ± 0.91	3 (3–4)	<0.001
Q2 (*p* < 0.001 **)	1.25 ± 0.52	1 (1–1)	

* Mann–Whitney U test; ** Shapiro–Wilk test.

**Table 2:** Distribution of patients with conventional dentures (Q1) and implant-supported dentures (Q2) according to responses for the 5 items.

Response	Q1		Q2	
	No.	%	No.	%
**Item 1: Have you had difficulty chewing any foods because of problems with your mouth, dentures, or jaw?**				
Never	1	1.9%	30	57.7%
Almost Never	2	3.8%	13	25.0%
Occasionally	16	30.8%	8	15.4%
Often	22	42.3%	1	1.9%
Very Often	11	21.2%	0	0.0%
*p*	<0.001			
**Item 2: Have you experienced painful aching in your mouth?**				
Never	2	3.8%	40	76.9%
Almost Never	14	26.9%	9	17.3%
Occasionally	30	57.7%	2	3.8%
Often	5	9.6%	1	1.9%
Very Often	1	1.9%	0	0.0%
*p*	<0.001			
**Item 3: Have you felt uncomfortable about the appearance of your mouth, dentures, or jaws?**				
Never	2	3.8%	39	75%
Almost Never	7	13.5%	9	17.3%
Occasionally	20	38.5%	4	7.7%
Often	16	30.8%	0	0.0%
Very Often	7	13.5%	0	0.0%
*p*	<0.001			
**Item 4: Have you noticed a reduced sense of taste due to problems with your mouth, dentures, or jaws?**				
Never	1	1.9%	48	92.3%
Almost Never	2	3.8%	3	5.8%
Occasionally	8	15.4%	1	1.9%
Often	23	44.2%	0	0.0%
Very Often	18	34.6%	0	0.0%
*p*	<0.001			
**Item 5: Have you had difficulty performing your usual activities due to problems with your mouth, dentures, or jaws?**				
Never	2	3.8%	41	78.8%
Almost Never	5	9.6%	9	17.3%
Occasionally	27	51.9%	2	3.8%
Often	13	25.0%	0	0.0%
Very Often	5	9.6%	0	0.0%
*p*	<0.001			

**Table 3:** Distribution of patients with conventional dentures (Q1) and implant-supported dentures (Q2) according to gender and responses to items.

Response	Q1				Q2			
	Female		Male		Female		Male	
	No.	%	No.	%	No.	%	No.	%
**Item 1: Have you had difficulty chewing any foods because of problems with your mouth, dentures, or jaw?**								
Never	0	0.0%	1	3.7%	15	60.0%	15	55.5%
Almost Never	1	4.0%	1	3.7%	5	20.0%	7	25.9%
Occasionally	6	24.0%	10	37.0%	5	20.0%	3	11.1%
Often	14	56.0%	8	29.6%	0	0.0%	2	7.5%
Very Often	4	16.0%	7	25.9%	0	0.0%	0	0.0%
*p*	0.282				0.421			
**Item 2: Have you experienced painful aching in your mouth?**								
Never	2	8%	0	0%	19	76.0%	20	74.0%
Almost Never	6	24%	8	29.6%	5	20.0%	5	18.5%
Occasionally	15	60%	15	55.6%	0	0.0%	2	7.5%
Often	1	4%	4	14.8%	1	4.0%	0	0.0%
Very Often	1	4%	0	0%	0	0.0%	0	0.0%
*p*	0.292				0.522			
**Item 3: Have you felt uncomfortable about the appearance of your mouth, dentures, or jaws?**								
Never	1	4.0%	1	3.7%	21	84.0%	18	66.6%
Almost Never	1	4.0%	6	22.2%	3	12.0%	6	22.2%
Occasionally	11	44.0%	9	33.3%	1	4.0%	3	11.2%
Often	8	32.0%	8	29.6%	0	0.0%	0	0.0%
Very Often	4	16.0%	3	11.1%	0	0.0%	0	0.0%
*p*	0.414				0.508			
**Item 4: Have you noticed a reduced sense of taste due to problems with your mouth, dentures, or jaws?**								
Never	0	0.0%	1	3.7%	24	96.0%	23	85.2%
Almost Never	1	4.0%	1	3.7%	1	4.0%	2	7.4%
Occasionally	4	16.0%	4	14.8%	0	0.0%	2	7.4%
Often	12	48.0%	11	40.7%	0	0.0%	0	0.0%
Very Often	8	32.0%	10	37%	0	0.0%	0	0.0%
*p*	0.981				0.797			
**Item 5: Have you had difficulty performing your usual activities due to problems with your mouth, dentures, or jaws?**								
Never	1	4.0%	1	3.7%	21	84.0%	20	74.1%
Almost Never	2	8.0%	3	11.1%	3	12.0%	6	22.2%
Occasionally	14	56.0%	13	48.1%	1	4.0%	1	3.7%
Often	6	24.0%	7	25.9%	0	0.0%	0	0.0%
Very Often	2	8.0%	3	11.1%	0	0.0%	0	0.0%
*p*	1.000				0.858			

**Table 4:** Distribution of patients with conventional dentures (Q1) and implant-supported dentures (Q2) according to age and responses to items.

Response	Q1		Q2	
	Mean Age ± SD	Median Age (IQR)	Mean Age ± SD	Median Age (IQR)
**Item 1: Have you had difficulty chewing any foods because of problems with your mouth, dentures, or jaw?**				
Never (*p* = - **)	70	70	60.33 ± 9.6	62 (51.5–68)
Almost Never (*p* = - **)	57 ± 12.72	57 (48–66)	58.08 ± 7.34	59 (51–64.5)
Occasionally (*p* = 0.203 **)	60.94 ± 7.31	61.5 (57.5–64.5)	58 ± 11.7	60.5 (45.75–67)
Often (*p* = 0.452 **)	62 ± 8.35	63.5 (58–66)	56	56
Very Often (*p* = 0.221 **)	56.91 ± 12.81	61 (44.5–65)	-	-
*p* *	0.562		0.750	
**Item 2: Have you experienced painful aching in your mouth?**				
Never (*p* = - **)	51.5 ± 14.85	51.5 (41–62)	59.75 ± 9.57	61.5 (51–67)
Almost Never (*p* = 0.008 **)	60.57 ± 8.89	61 (58–63)	56.44 ± 8.09	55 (53–62)
Occasionally (*p* = 0.807 **)	62.47 ± 8.16	64 (58–69)	62.5 ± 12.02	62.5 (54–71)
Often (*p* = 0.047 **)	55.4 ± 12.34	63 (42–63)	62	62
Very Often (*p* = - **)	47	47	-	-
*p* *	0.232		0.688	
**Item 3: Have you felt uncomfortable about the appearance of your mouth, dentures, or jaws?**				
Never (*p* = - **)	72 ± 2.82	72 (70–74)	59.54 ± 9.31	62 (50–67)
Almost Never (*p* = 0.177 **)	61.14 ± 3.89	61 (57.5–64.5)	56.44 ± 9.9	54 (51–62.5)
Occasionally (*p* = 0.005 **)	60.7 ± 9.23	63 (59.5–66.5)	63.75 ± 6.29	63 (58.25–70)
Often (*p* = 0.622 **)	60.06 ± 8.91	61.5 (55–64.5)	-	-
Very Often (*p* = 0.156 **)	57.43 ± 13.74	60 (44.5–69)	-	-
*p* *	0.264		0.355	
**Item 4: Have you noticed a reduced sense of taste due to problems with your mouth, dentures, or jaws?**				
Never (*p* = - **)	70	70	59.65 ± 9.21	61.5 (52.5–67)
Almost Never (*p* = - **)	53.5 ± 17.67	53.5 (41–66)	54.67 ± 12.01	54 (48.5–60.5)
Occasionally (*p* = 0.242 **)	59.5 ± 10.87	60 (57–65.5)	58	58
Often (*p* = 0.112 **)	61.83 ± 7.7	63 (60–64.5)	-	-
Very Often (*p* = 0.123 **)	59.67 ± 9.96	62.5 (54–67)	-	-
*p* *	0.647		0.688	
**Item 5: Have you had difficulty performing your usual activities due to problems with your mouth, dentures, or jaws?**				
Never (*p* = - **)	55.5 ± 20.5	55.5 (41–70)	58.61 ± 9.77	60 (49–65)
Almost Never (*p* = 0.526 **)	64.4 ± 6.42	65 (59–66)	61 ± 6.55	61 (54–67)
Occasionally (*p* = 0.112 **)	62.78 ± 6.22	63 (58.5–64.5)	66.5 ± 6.36	66.5 (62–71)
Often (*p* = 0.063 **)	57.15 ± 12.4	62 (45–66)	-	-
Very Often (*p* = 0.460 **)	55.6 ± 10.14	60 (47–61)	-	-
*p* *	0.676		0.390	

* Mann–Whitney U test; ** Shapiro–Wilk test.

## Data Availability

The data presented in this study are available upon request from the corresponding author due to ethical reasons.
